# Uncertainty Quantification in Constitutive Models of Highway Bridge Components: Seismic Bars and Elastomeric Bearings

**DOI:** 10.3390/ma16051792

**Published:** 2023-02-22

**Authors:** Francisco J. Pinto, José Toledo, Matías Birrell, Ramiro Bazáez, Francisco Hernández, Rodrigo Astroza

**Affiliations:** 1 Facultad de Ingeniería y Ciencias Aplicadas, Universidad de los Andes, Santiago 7620001, Chile; 2 Techint Engineering and Construction, Santiago 7560742, Chile; 3 Departamento de Obras Civiles, Universidad Técnica Federico Santa María, Valparaíso 2390123, Chile; 4 Department of Civil Engineering, Universidad de Chile, Santiago 8370449, Chile

**Keywords:** Bayesian estimation, bridge components, constitutive material models

## Abstract

Bridges are essential structures in the logistic chain of countries, making it critical to design them to be as resilient as possible. One way to achieve this is through performance-based seismic design (PBSD), which involves using nonlinear Finite Element (FE) models to predict the response and potential damage of different structural components under earthquake excitations. Nonlinear FE models need accurate constitutive models of material and components. Among them, seismic bars and laminated elastomeric bearings play an important role in a bridge’s response to earthquakes; therefore, properly validated and calibrated models should be proposed. Only default parameter values from the early development of the constitutive models widely used by researchers and practitioners for these components tend to be used, and low identifiability of its governing parameters and the high cost of generating reliable experimental data have prevented a thorough probabilistic characterization of their model parameters. To address this issue, this study implements a Bayesian probabilistic framework using Sequential Monte Carlo (SMC) for updating the parameters of constitutive models of seismic bars and elastomeric bearings and proposes joint probability density functions (PDF) for the most influential parameters. The framework is based on actual data from comprehensive experimental campaigns. The PDFs are obtained from independent tests conducted on different seismic bars and elastomeric bearings, to then consolidate all the information in a single PDF for each modeling parameter by means of the conflation methodology, where the mean, coefficient of variation, and correlation between calibrated parameters are obtained for each bridge component. Finally, findings show that the incorporation of model parameter uncertainty through a probabilistic framework will allow for a more accurate prediction of the response of bridges under strong earthquakes.

## 1. Introduction

New design and analysis methodologies for structures, such as performance-based seismic design (PBSD), require detailed finite element (FE) models to accurately capture the nonlinear behavior of systems under earthquake excitations [1,2,3,4]. In the past few decades, academic and engineering communities have carried out cutting-edge research regarding the performance and modeling of bridges and their components under severe ground motions. For example, Hube et al. [5] evaluated the damage in Chilean bridges during the Maule 2010 earthquake and studied the impact of diaphragms in the seismic performance of such bridges. Elnashai et al. [6], in their investigation of typical failures produced in Chilean bridges, showed the significance of using shear keys as sacrificial elements to reduce damage to columns and beams. Wilches et al. [7] discussed, statistically, the importance of bridge components on the transverse and vertical responses of typical Chilean bridges, highlighting the importance of seismic bars (SBs) in reducing the probability of deck uplift. Aldea et al. [8] emphasized the necessity of proper modeling vertical components, such as laminated elastomeric bearings (EBs), to avoid underestimating displacement demands in multi-span, simply supported, reinforced concrete (RC) bridges. These studies have shown the importance of bridge components (e.g., SBs and EBs) in the response of bridges and the need to accurately model these components to develop detailed finite element models that can provide crucial insights into the effectiveness of methodologies, such as performance-based seismic design (PBSD), in bridge engineering.

**Figure 1:**
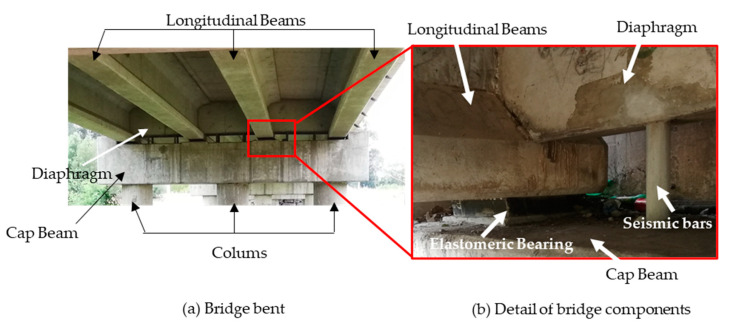
Details of a bridge’s components at the bridge bent.

SBs are a vertical element made of ductile steel bars that connect the superstructure slab to its abutment or cap beams, as shown in Figure 1. These bars resist vertical forces and provide lateral stiffness to the deck during a large lateral displacement of the superstructure such as during earthquake motions [7,9]. On the other hand, EBs support the longitudinal beams (as shown in Figure 1). These bearings are designed to meet service-level requirements by sustaining vertical dead-loads and accommodating horizontal movements of the superstructure due to service actions and eventual actions such as ground motion. The behavior of EBs varies based on their installation method, which separates them into two main categories: unanchored EBs (UEBs) and anchored EBs (AEBs). The AEBs are those having the top and bottom surfaces securely anchored to the superstructure and substructure, while UEBs are usually placed directly between the superstructure and substructure without any connection other than friction at the contact surfaces. The installation method affects the behavior of EBs under dynamic lateral loads, with the surface friction and rubber shear stiffness providing lateral resistance and deformation in UEBs, while AEBs derive their lateral strength from the full shear stiffness and strength of the bearing [10].

Since the performance of SBs and EBs play a fundamental role in the response of bridges subject to earthquakes, several studies have focused on the structural behavior of these components as well as their numerical modeling. Aviram et al. [11] and Steelman et al. [12] have proposed EB numerical models that utilize elastic perfectly plastic and linear elastic behavior, respectively. Filipov et al. [13] developed an analytical model for EB that accounts for two force peaks in the hysteretic behavior—the first one referring to when it starts sliding and the other when it stops sliding. Kostantinidis et al. [14] suggested that yielding of EB occurs at shear deformations ranging from 150–225% of the rubber height. Rubilar [15] conducted an extensive experimental campaign to develop a two-dimensional nonlinear model for the seismic response of overpasses, including a constitutive model for unanchored elastomeric bearings. Martinez et al. [9] proposed a constitutive model for SBs based on experimental data.

The implementation in the engineering practice of state-of-the-art constitutive models for SBs and EBs, as previously mentioned, is developed under assumption of the correct values of the model parameters used in their formulation. Nevertheless, calibrating these constitutive models to account for accurate and precise numerical responses of bridges is essential due to the presence of various sources of uncertainty, such as modeling errors, parameter uncertainty, and measurement noise [16]. A probabilistic approach, such as the Bayesian framework for model updating (e.g., [17,18]), is, therefore, recommended for calibration of bridge components. This method allows the incorporation of different sources of uncertainty and subsequently provides the most probable model parameter values based on recorded data. The result is the generation of posterior probability density functions (PDFs), which give a probabilistic description of the model parameters.

This study proposes joint PDFs for the state-of-the-art constitutive model of SBs and EBs using comprehensive datasets from experimental campaigns conducted by Rubilar [15], Martinez [9], and test results reported herein. The posterior distributions for each dataset are obtained through a conflation approach and grouped by bridge component, allowing for efficient sampling of the model parameters. The calibrated results show consistent parameter correlations among datasets, which are condensed in a proposed correlation matrix for general sampling of SB and EB model parameters. By means of this approach, more reliable fragility curves of components and bridge structures can be generated, making use of more robust methodologies and seismic databases, and accounting for the model parameter uncertainties present in the problem of fragility assessment of bridges during earthquakes.

## 2. Experimental Data

### 2.1. Seismic Bars (SBs)

The experimental data used to calibrate model parameters were collected from tests conducted by Martinez et al. [9]. These tests were designed to simulate the behavior of SBs in bridges with (SBs-WD) and without (SBs-WOD) diaphragms. The experimental setup for the tests consisted of a reinforced concrete (RC) block at the bottom representing the bent cap of the substructure, a RC block at the top representing the diaphragm or slab, and two seismic bars connecting both blocks. The clear height between blocks (*h_l_*) was the distance from the bottom of the bent cap to the slab of the bridges. Four specimens were used in the study: WD1, WD2, WOD1, and WOD2 with different *h_l_*; see Table 1.

The SBs were 16 mm in diameter and made of A440–280H steel with a yield and an ultimate strength of 280 MPa and 440 MPa, respectively. The reinforcing bars of RC elements were made of A630–420H steel with a yield and an ultimate strength of 420 MPa and 630 MPa, respectively. The concrete used in the specimens had a maximum compress strength of 20 MPa and a maximum aggregate size of 20 mm. More information on the setup for the specimens can be found in Martinez et al. [9].

### 2.2. Elastomeric Bearings (EBs)

#### 2.2.1. Unanchored Elastomeric Bearing (UEB)

The experimental data used in this study to calibrate the constitutive model of unanchored (or unbonded) elastomeric bearings (UEB) were collected by Rubilar [15] in a campaign focused on characterizing the experimental behavior of these bearings under cyclic and monotonic displacements. In this campaign, six identical UEBs with dimensions of 400 mm × 500 mm × 90 mm, reinforced with 3 mm thick steel plates and a rubber height of 72 mm, were tested under vertical compression loads of 400 kN and 600 kN. The bearings were subjected to incremental lateral displacements of 9 mm, 22.5 mm, 45 mm, 90 mm, 135 mm, and 144 mm, corresponding to shear deformations of 10%, 25%, 50%, 100%, 150%, and 160%, respectively. These tests were conducted at the testing speeds provided in Table 2. During the test, the bearings were placed between two RC blocks with the lower block having a rough surface to simulate the leveling mortar in real beams and the upper block having a smooth surface to represent the bottom web of a prestressed RC girder.

#### 2.2.2. Anchored Elastomeric Bearing (AEB)

For the analysis of anchored elastomeric bearings (AEB), a database of 22 tests conducted on AEB is employed in this study. Table 3 provides the relevant information for the tested bearings that are used in the uncertainty quantification process.

## 3. Constitutive Models

### 3.1. Seismic Bars (SBs)

**Figure 2:**
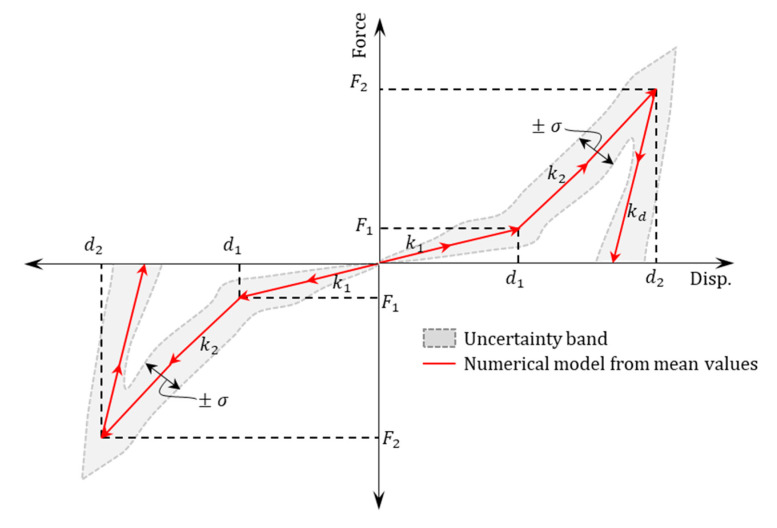
Load-displacement model considered for SBs.

The constitutive model for SBs is based on the model proposed by Martinez et al. [9], for both cases SBs-WD and SBs-WOD. It is built on the load-displacement relationship shown in Figure 2, which includes two points and an unloading stiffness. The displacement of the first point denoted by $d_{1}$ can be determined using Equation (1), and defines the transition from a predominantly flexural behavior to a tensile behavior, while the displacement of the second point denoted by $d_{2}$ (Equation (2)) corresponds to an approximation of the maximum displacement ($d_{max}$) observed in the specimen tests. $g_{1}$ and $g_{2}$ are two dimensionless parameters controlling the amplitude of $d_{1}$ and $d_{2}$, respectively.

(1) $$d_{1}=g_{1}*h_{1}$$

(2) $$d_{2}=\left\{\begin{array}{c} \;\;\;h_{l},\;\;\;\;\;\;\;\;SB-WD\\ g_{2}*h_{l},\;\;\;\;\;SB-WOD \end{array}\right.$$

The lateral force at each point on the hysteretic curve ($F_{1}$ and $F_{2}$) of SBs can be calculated using Equation (3), where i takes the values 1 and 2 for each point. In this equation, the actual yield stress ($f_{y}^{*}$) is estimated as 1.2 times the nominal yield stress of steel $\left(f_{y_{nominal}}\right)$ and $A_{sb}$ corresponds to the total transverse area of SBs. The dimensionless factor, $\gamma_{i}$, is used to estimate the lateral forces on the hysteretic curve of SBs ($F_{1}$ and $F_{2}$) and considers the ratio of lateral stress in SBs to the yield stress of steel and the rotation of SBs during loading. The values of $\gamma_{i}$ are provided in Table 4 according to Martinez et al. [9]

(3) $$F_{i}=\gamma_{i}*f_{y}^{*}*A_{sb}$$

The unloading stiffness ($k_{d}$) is calculated using Equation (4). It is determined by multiplying the second loading stiffness ($k_{2}$) by a factor that depends on the absence or presence of the diaphragm.

(4) $$k_{d}=\left\{\begin{array}{c} 20*k_{2},\;\;\;\;SB-WD\\ \;\;\;15*k_{2},\;\;\;\;\;SB-WOD \end{array}\right.\;$$

The behavior of seismic bars is modeled in OpenSees [19] using a *zero-length* element with transverse and longitudinal behavior adjusted to the combination of the *uniaxialMaterial Hysteretic* and *uniaxialMaterial MinMax* materials. The former generates a bilinear hysteretic curve with the three points in the envelope and the latter allows for the definition of displacement under which the bridge component remains without failure.

### 3.2. Elastomeric Bearings

#### 3.2.1. Unanchored Elastomeric Bearing (UEB)

**Figure 3:**
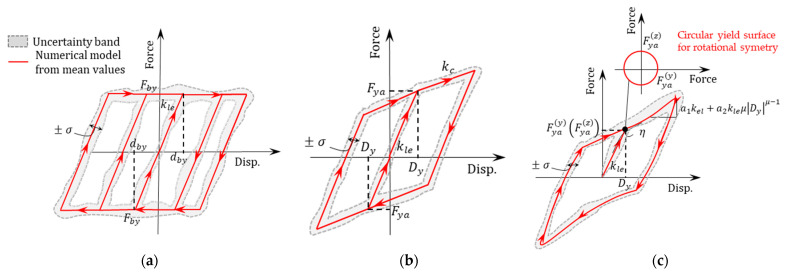
(**a**) Elastic perfectly plastic load-displacement model for UEB; (**b**) elastic plastic load-displacement model for UEB; and (**c**) Bouc–Wen model representing the lateral load-displacement relationship for AEB.

The constitutive model for UEB, as proposed by Rubilar et al. [15], is based on the assumption of perfect elastoplastic behavior that is characterized by the hysteretic relationship between the lateral stiffness ($k_{le}$) and the yield force ($F_{by})\;$of the bearing. This relationship is depicted in Figure 3a.

$k_{le}$ can be calculated using Equation (5). It is obtained by the product of the shear modulus of the bearing ($G_{eb}$) by the ratio of the area of the bearing ($A_{eb}$) to the height of the rubber ($H_{r}$).

(5) $$k_{le}=G_{eb}*A_{eb}/H_{r}$$

$F_{by}$ dependents on the rubber friction coefficient ($\mu_{e}$) and the normal force on the UEB and can be computed using Equation (6).

(6) $$F_{by}=\mu_{e}*\sigma_{cd}*A_{eb}$$

where $\sigma_{cd}$ is the design compressive stress, which is 1.15 times the normal stress $\sigma_{c}$ due to the dead load on the bearing. 

OpenSees [19] software is used to model the lateral response of UEB by means of a zero-length element, where the transverse and longitudinal behavior of the model is determined by combining the *uniaxialMaterial Steel01* and *uniaxialMaterial MinMax* materials. The *uniaxialMaterial Steel01* material generates a bilinear curve, while the *uniaxialMaterial MinMax* material allows the imposition of the limit displacements under which the bridge components work.

#### 3.2.2. Anchored Elastomeric Bearing (AEB)

The behavior of AEB can be achieved by means of two approaches as proposed in [7,20]. The first approach (AEB-model 1) uses an elastic-plastic model and it is defined by two fundamental slopes (see Figure 3b): the loading and unloading slope ($k_{le}$) and the slope after the yield point ($k_{c}$). $k_{le}$ can be calculated using Equation (5). This value has a similar definition to that of UEB. The yield deformation ($D_{y}$) and failure deformation ($d_{f}$) of AEB are then determined based on the height of the rubber ($H_{r}$), as expressed in Equations (7) and (8), respectively:

(7) $$D_{y}=0.05*H_{r}$$

(8) $$d_{f}=1.5*H_{r}$$

Using these parameters, the failure force ($F_{fa}$) of AEB can be calculated using Equation (9):

(9) $$F_{fa}=F_{ya}+k_{c}*\left(d_{f}-D_{y}\right)$$

where $k_{c}$ is defined as in Equation (10) and $F_{ya}$ is the yield force.

(10) $$k_{c}=F_{ya}/D_{y}\;$$

The AEB-model 1 can be implemented in OpenSees [19] using a *zero-length* element with transverse and longitudinal actions that are adjusted to the combination of the *uniaxialMaterial Hysteretic* and *uniaxialMaterial MinMax* materials. The *uniaxialMaterial Hysteretic* material allow for the definition of the two fundamental slopes of the constitutive model, $k_{c}$ and $k_{le}$. The *uniaxialMaterial MinMax* material is used to incorporate the maximum displacement under which the bridge component functions.

The second approach (AEB-model 2) uses the constitutive model proposed by Bouc–Wen [21,22] (see Figure 3c). This model can be implemented in OpenSees [19] using the *ElastomericBearingBoucWen* element, which in turn requires the definition of models representing the vertical and rotational behavior of the elastomeric bearings. For this purpose, a *zero-length* element is defined, where the vertical and rotational behavior is represented by a *uniaxialMaterial Hysteretic* material. The vertical behavior is described by the vertical stiffness of the bearing ($k_{ve}$), Equation (11).

(11) $$k_{ve}=E_{r}*A_{eb}/H_{r}\;$$

where $E_{r}$ represents the elastic modulus of rubber and is a function of $G_{eb}$ and the shape coefficient of the elastomeric bearing ($S_{b})$, as shown in Equation (12).

(12) $$E_{r}=4.8*G_{eb}*S_{b}^{2}\;$$

$S_{b}$ can be calculated using Equation (13), which relates it to the thickness ($e_{r}$), length ($L_{r}$), and width ($B_{r}$) of the rubber.

(13) $$S_{e}=A_{eb}/\left(2*e_{r}*\left(L_{r}+B_{r}\right)\right)\;$$

The rotational behavior of the elastomeric bearing can then be described using Equation (14), which represents the rotational stiffness ($k_{\theta e}{}_{\;}$) of the bearing. This equation involves the computation of the plate rotational inertia ($I_{e}$) using Equation (15).

(14) $$k_{\theta e}=0.5*E_{r}*I_{e}/H_{r}\;$$

(15) $$I_{e}=A_{eb}*L_{r}^{3}/12\;$$

In addition, the use of the Bouc–Wen model in OpenSees [19] requires the definition of the initial elastic stiffness in the local direction of shear ($k_{c}$) as in Equation (10), the post-yield stiffness ratio of the linear hardening component ($a_{1}$) as expressed in Equation (16), and the dimensionless quantities controlling the scale and shape of the hysteresis loop shown in Table 5. These dimensionless parameters are defined according to the agreement between the predicted response and experimental data during the calibration process.

(16) $$a_{1}=k_{le}/k_{c}\;$$

## 4. Bayesian Parameter Estimation

Bayesian updating of constitutive models of bridge components is a process that involves using experimental data to properly calibrate and improve the accuracy of these models; see [17,18]. This calibration process allows for the obtainment of the PDFs of the constitutive model parameters for SBs and UEB and AEB components. Following Bayesian updating, the uncertainty quantified in constitutive model parameters of a bridge’s components can then be propagated through numerical FE models, leading to more comprehensive probabilistic seismic analysis of bridges.

To address the problem of calibrating constitutive models for bridge components in this study, a sampling method called Sequential Monte Carlo (SMC) is used. This method combines techniques such as importance sampling, tempering, and Markov Chain Monte Carlo (MCMC) to efficiently explore the parameter space and determine the posterior PDFs of the model parameters. One of the advantages of using the SMC method is its ability to accurately determine the PDFs in cases where the response has multiple peaks, which can occur when studying nonlinear finite element models [23,24].

### 4.1. Bayesian Inversion

In this work, Bayesian inversion is used to infer a vector of model parameters θ that define a specific model class M from a set of observed data $y_{obs}$. The data consist of $N_{obs}$ observations, each with $N_{out}$ points. The posterior distribution of θ is defined from Bayes’ theorem as in Equation (17).

(17) $$P\left(\theta |y_{obs}\right)=\frac{P\left(y_{obs}|\theta \right)P\left(\theta \right)}{P\left(y_{obs}\right)}$$

where $P(\theta |y_{obs})$ is the posterior distribution of θ, $P\left(\theta \right)$ is the prior distribution of θ, $P(y_{obs}|\theta )$ is the likelihood of M predicting $y_{obs}$ through θ, and $P\left(y_{obs}\right)=P(y_{obs}|M)=\int_{-\infty }^{\infty }P\left(y_{obs}|\theta \right)P\left(\theta \right)d\theta$ is the model evidence and normalizes the posterior such that it integrates to 1.

The posterior distribution combines prior beliefs about the value of θ with information inferred from the data through the likelihood function. If we assume independence between observations, the likelihood function can be written as in Equation (18).

(18) $$P\left(y_{obs}|\theta \right)=\prod_{k=1}^{N_{obs}}P\left(y_{obs,k}|\theta \right)$$

As a consequence, the posterior distribution represents an “updated” belief by combining the likelihood inferred from experimental data with the prior beliefs about parameter values. It is useful to consider different types of prior distributions, as they can provide varying amounts of information about θ. The likelihood function shows that the number of observations can have an impact on the posterior distribution, particularly in relation to the variability between observations. For example, a larger number of observations may result in a decrease in the marginal contribution of each observation to the posterior, and larger variability in the observations may result in a wider posterior. Therefore, it is important to carefully select an adequate dataset for updating. The likelihood of data is often assumed to follow a normal distribution with a variance term $\;\sigma^{2}$, referred to as model discrepancy. This term represents non-modeled sources of error in the model M and measurement noise. It can be modeled as a random variable or considered constant. It is important to assess the sensitivity of the posterior to the model discrepancy. For example, a larger value of σ may lead to less restrictive posterior convergence, but also more uncertain predictions.

### 4.2. Markov Chain Monte Carlo (MCMC)

In practice, solving the expression (17) analytically is not feasible for computer models, so a simulation approach such as MCMC is used. This method helps to sample multiple chains from the posterior distribution by proposing updates to the prior distribution. At each step, the proposed sample’s posterior distribution is evaluated and accepted or rejected based on the model’s fit to the observed data. Multiple sampling algorithms are available for MCMC; in this work, the Metropolis–Hastings (MH) [25] is used. Therefore, the MCMC allows for the obtainment of parameter posterior PDFs by the sampling of parameter prior PDFs and the likelihood of observed data conditioned to sampled parameters. The posteriors resulting from Bayesian estimation quantify the uncertainty in the model’s response. The implementation of Bayes’ rule for parameter estimation is further detailed through reviews of Monte Carlo methods and their implementation available in Kroese et al. [26] and Wagner et al. [27].

### 4.3. Tempering

This methodology focuses mainly on the use of an auxiliary temperature parameter to control the convergence progress of the samples generated using MCMC. This parameter generates the posterior approach in Equation (19).

(19) $$p{\left(\theta |y_{obs}\right)}_{\beta }\propto p{\left(y_{obs}|\theta \right)}^{\beta }p\left(\theta \right)\;$$

If β=0, the resultant is the prior PDF, but if β=1, the resultant is the posterior PDF. By means of the following steps, the methodology behind SMC is executed [23,28]:

1. Sample each parameter θ from a prior distribution.
2. Simulate a dataset $y^{*}$ using a function that takes the parameters and returns the predicted data ($y_{0}$), considering the dimension of the observed data.
3. Compare $y^{*}$ and $y_{0}$ using the distance function and a tolerance threshold value.
4. When β=1, the distance function value is less than the threshold value; if this tolerance value is sufficiently small, the distribution obtained will be a good approximation for the posterior $P\left(\theta |y_{0}\right).$

### 4.4. Convergence Criteria

One of the main challenges in statistical analysis is ensuring that the obtained results accurately reflect the true underlying relationships in the data. This is especially important when using Monte Carlo techniques, such as MCMC, which involve repeatedly sampling from a probability distribution to estimate statistical parameters. One way to assess the accuracy of the results is to calculate the effective sample size (ESS). There are two main approaches for calculating ESS: univariate and multivariate. The univariate approach calculates ESS values for each parameter individually, ignoring any correlations between parameters. This can be useful in cases where the parameters are independent of each other. However, in many real-world situations, there are correlations between parameters that need to be taken into account. This is where the multivariate effective sample size (mESS) method comes in. This method considers the correlations between parameters, which can provide a more accurate result [29]. This concept is defined below by means of Equation (20).

(20) $$mESS=n{\left(\frac{\left|\Lambda \right|}{\left|\Sigma \right|}\right)}^{1/p\;}\;$$

where Λ represents the covariance of the posterior distribution, while Σ represents the asymptotic covariance matrix in the Central Limit Theorem for a Markov chain. The symbol *p* represents the number of parameters, and the $\left|\cdot \right|$ operator denotes the determinant. 

To accurately calibrate the parameters of nonlinear FE models, the methodology proposed by Vats et al. [29] and outlined in Equation (21) can be used. Specifically, the minimum required value of mESS in order to guarantee convergence must be determined. This approach has been widely discussed and is a reliable method for solving this general problem.

(21) $$mESS\geq \frac{2^{\frac{2}{p}}\pi }{{\left(p\Gamma \left(\frac{p}{2}\right)\right)}^{\frac{2}{p}}\;}\;\frac{\chi_{1-\alpha ,p}^{2}}{\xi^{2}}$$

where, α denotes the level used for constructing confidence intervals, and ξ stands for the desired fractional error of the Monte Carlo method compared to the error of the posterior. The symbol Γ represents the gamma function and χ is the inverse chi-squared distribution. 

### 4.5. Conflation Procedure

The conflation methodology is employed to combine the results of independent parameter calibrations [30]. This approach is an alternative to simply averaging the data or probabilities. Mathematically, conflation is represented by the symbol &, indicating that information from multiple PDFs is consolidated. In this scenario, since all parameters have a normal density function, Equation (22) is applicable.

(22) $$f\left(x\right)=\frac{f_{1}\left(x\right)f_{2}\left(x\right)\ldots f_{n}\left(x\right)}{\int_{-\infty }^{\infty }f_{1}\left(x\right)f_{2}\left(x\right)\ldots f_{n}\left(x\right)dy}$$

## 5. Results and Discussion

### 5.1. Calibration

This section applies the Bayesian updating procedure, as described in Section 4, to calibrate the model parameters for each dataset of SBs, UEB, and AEB. Using the MCMC algorithm, posterior distributions including marginal PDFs and parameter correlations for each bridge component are obtained. Then, these statistical results are combined using the conflation method to propose a single PDF that covers all datasets of each bridge component, from which predictions can be generated. One assumption made in this process is that the model’s predictive capacity is the same for each dataset, which is represented by a constant variance in the likelihood function. While newer methods have been proposed for estimating multi-response likelihood functions that do not make this assumption, it is preferred to use a simpler probabilistic model consisting of independent estimations for each dataset. This is because the constitutive models for the hysteretic behavior of SBs, UEB, and AEB are unidimensional and are expected to produce similar prediction errors across datasets. While a more sophisticated calibration scheme could be developed using recent advancements, the primary goal of proposing general use parameter PDFs for each bridge component can still be achieved using the standard method, as demonstrated by the good agreement between measured data and numerical response presented below.

The constitutive model parameters of each bridge component to be estimated are presented in Table 6. The other parameters of the constitutive models not included in Table 6 are geometric quantities or dependent on the test conditions, which implies that the uncertainty present does not significantly affect the model response. The prior PDFs were defined as normal in all cases based on available data for the bridge components, and they were selected such that the range of responses obtained from sampling covered the range of observed responses of all tests. The means of these prior PDFs were determined based on available statistical studies, and the COVs were chosen based on extreme values reported in the literature and the observed variance among tests. Table 6 presents the mean and coefficients of variation (COV) for the prior distributions employed for the predicted response and KDE matrix of the representative case discussed in Section 5.1.1 , Section 5.1.2 and Section 5.1.3. The MH [25] algorithm was employed for MCMC sampling, since it resulted in lower computation times than other alternatives such as Slice sampling [31]. 

To determine the number of iterations to be performed, the mESS proposed by Vats et al. [29] was computed for a confidence interval of 0.99 and relative precision of 0.1. For these values and the larger number of parameters in the constitutive models (6), the obtained minimum mESS was 1000 for each dataset. Convergence of the chains was assessed through the Gelman–Rubin estimator $\hat{R}$ [32] and cross-checked graphically through Kernel density estimation (KDE), for which traces include PDFs of the parameter and correlation ρ between the calibrated parameters. Additionally, statistics of interest from traces were computed, including posterior means μ, standard deviations σ, and 95% confidence intervals (i.e., percentiles 5% and 95%). Finally, posterior information was extracted from the combined samples obtained from the chains for each constitutive model for the bridge components, and the mESS for each dataset was computed to evaluate the termination condition (i.e., mESS larger than the minimum mESS computed as described above). 

#### 5.1.1. Seismic Bars (SBs)

**Figure 4:**
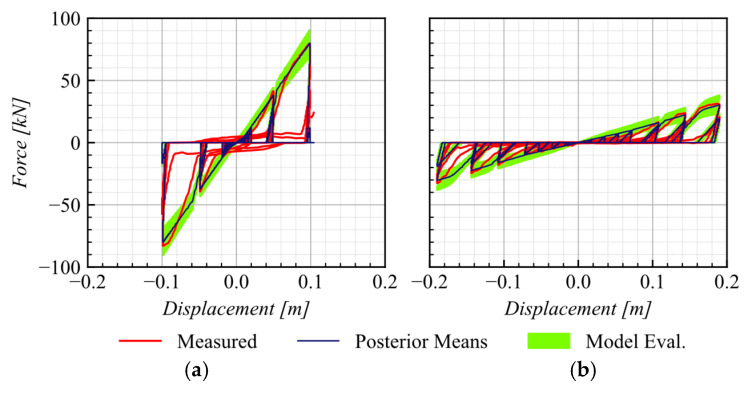
Comparison of the responses of (**a**) SBs-WD (WD2) and (**b**) SBs-WOD (WOD1) as tested by Martinez et al. (2017) with a calibrated response at posterior means, 5th and 95th percentiles.

Figure 4a SBs-WD (WD2) and Figure 4b SBs-WOD (WOD1) of SBs, as tested by Martinez et al. [9] with a calibrated response using posterior means as well as 5th and 95th percentiles, confirmed an excellent agreement between the experimental and predicted responses. 

**Figure 5:**
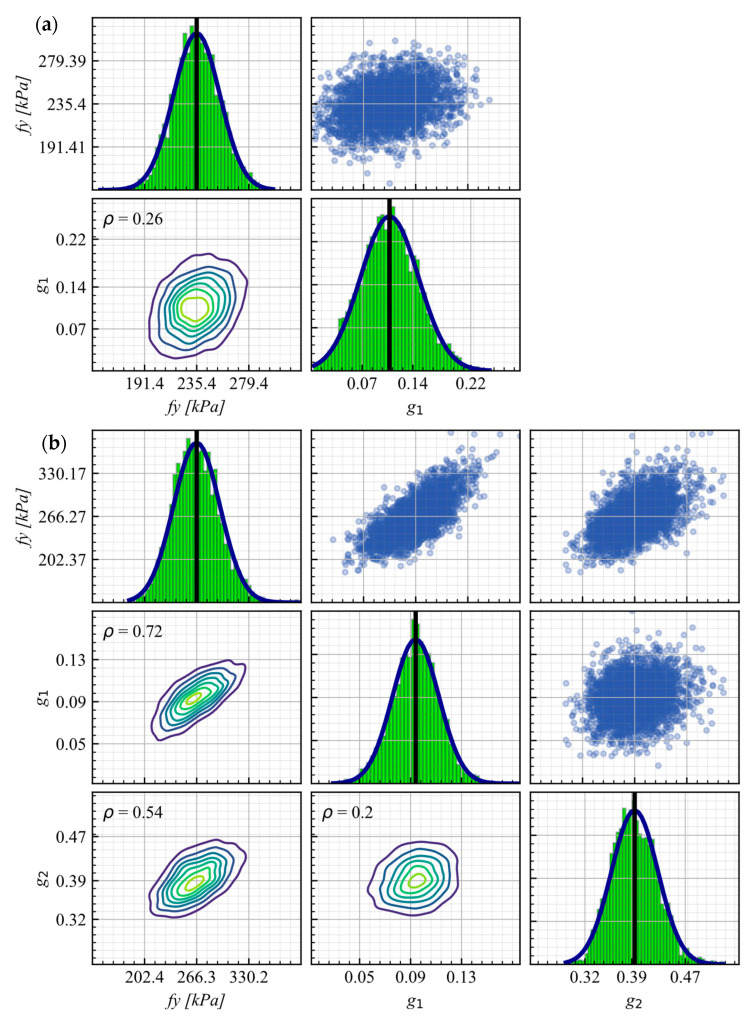
KDE matrix for (**a**) SBs-WD (WD2) and (**b**) SBs-WOD (WOD1).

The KDE matrices for SBs-WD (WD2) and SBs-WOD (WOD1), as representative cases, are presented in Figure 5a,b, respectively. Figure 5a, as well as MCMC traces for SBs-WD, show that the average correlation between $f_{y}$ and $g_{1}$ is relatively low (0.26) for SBs-WD. In contrast, Figure 5b implies that the average correlation between $f_{y}$ and the parameters $g_{1}$ and $g_{2}$ is high for SBs-WOD, with values of 0.72 and 0.54, respectively.

The low correlation between $f_{y}$ and $g_{1}$ in SB-WD is due to the presence of the diaphragm, which is expected to lead to a more controlled lateral displacement of the bridge. As a result, the SB-WD acts more like a tensor that controls the uplift of the bridge’s slab. In contrast, SB-WOD is designed to resist vertical forces (prevent uplift), while also providing lateral stiffness to the deck during large displacements. This leads to a greater dependence between $f_{y}$ and the dimensionless parameters ($g_{1}$ and $g_{2}$) that control the characteristic displacements that define the constitutive model of SB-WOD.

**Figure 6:**
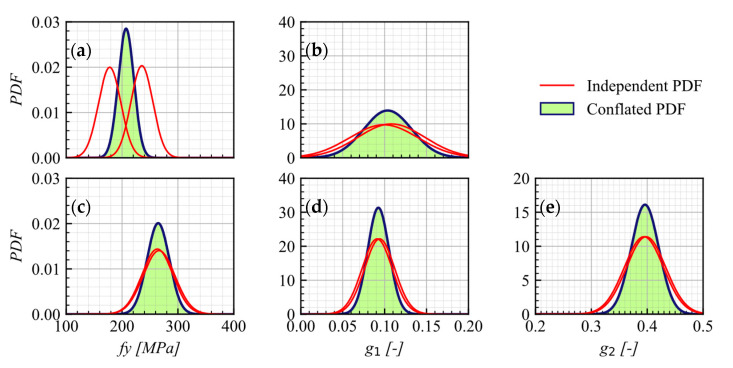
Conflated PDFs for $f_{y}$ (**a**) and $g_{1}$ (**b**) in the SBs-WD model, and conflated PDF for $f_{y}$ (**c**), $g_{1}$ (**d**), and $g_{2}$ (**e**) on in the SBs-WOD model.

After obtaining the calibrated parameters and their uncertainties, the methodology outlined in Section 4 was applied to merge the PDFs obtained from the calibration of independent experiments. This resulted in Figure 6, which illustrates the final PDFs of the constitutive models’ parameters for both SBs-WD and SBs-WOD models. According to the results shown in Figure 6a,c, the mean values of $f_{y}$ for SBs-WD and SBs-WOD models are 206.4 MPa and 264.6 MPa, respectively. Figure 6b shows that the mean value of $g_{1}$ for the SBs-WD model is 0.104, while it is 0.092 for the SBs-WOD model as shown in Figure 6d. Additionally, the mean value of $g_{2}$ for the SBs-WOD model is 0.395 as shown in Figure 6e. The values of $g_{1}$ and $g_{2}$ for SB-WOD are consistent with the expected values reported by Martinez et al. [9] of 0.1 and 0.35, respectively. The consistency of $g_{1}$ for both SBs-WD and SBs-WOD models is also in line with the expectation that it should be similar for both models.

#### 5.1.2. UEB

**Figure 7:**
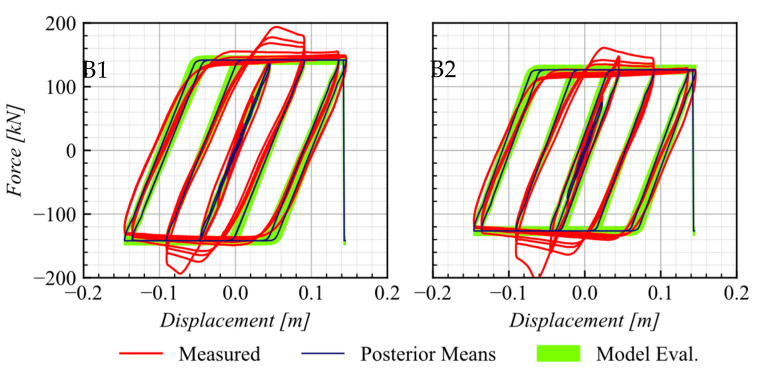
Comparison of the response of UEB as tested by Rubilar et al. (2015) with calibrated response at 5th and 95th percentiles.

A comparison of the predicted response from the calibration process and the experimental data from the B1 and B2 test (see Table 2), conducted under a compression load of 400 kN, is presented in Figure 7. The predicted responses using posterior means and the 5th and 95th percentiles show a strong agreement with the response measured during the experimental test.

**Figure 8:**
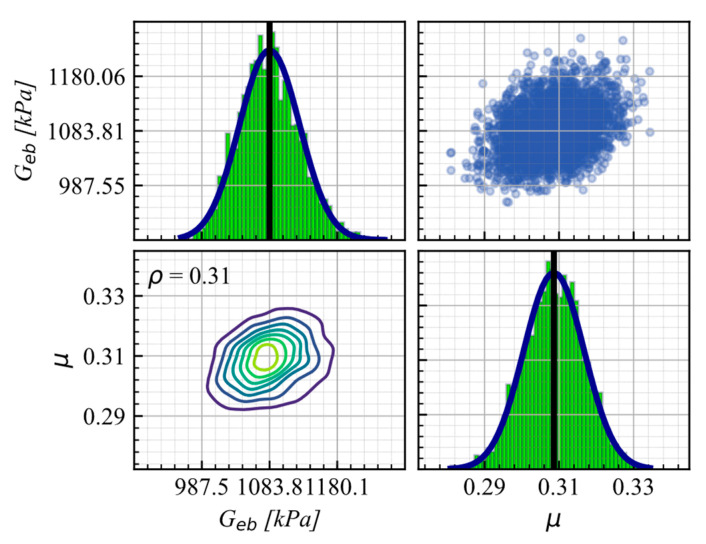
UEB KDE matrix for B1-C2 as the representative case.

Figure 8 shows the KDE Matrix for B1 under the compression load of the 400 kN test, as the representative case, displaying the PDFs and the correlation of 0.31 between the calibrated parameters. The overall average correlation of the parameters across all cases was 0.41.

**Figure 9:**
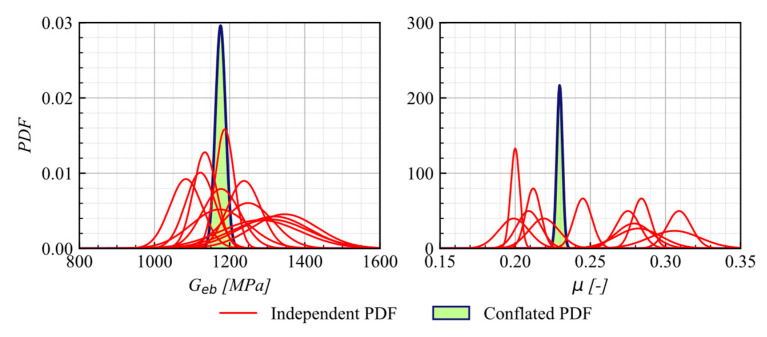
Conflated PDFs for G and μ on the unanchored elastomeric bearing model.

The posterior PDFs obtained by conflation of all independent experiments used as input are shown in Figure 9. From conflation, it is observed that the mean value of G and μ is 1176 kN/m^2^ and 0.23, respectively. The predicted mean value of G falls within the range 981–1275 kN/m^2^, suggested by AASHTO (2012) [33], while μ shows a predicted mean value similar to those recommend in AASHTO (2017) [20] of 0.2.

#### 5.1.3. AEB

The two modeling approaches detailed in Section 3.2.2 are used to calibrate the AEB numerical response. The main difference in the calibration between these approaches is the complexity in the number of calibrated parameters. The first approach, AEB-model 1, requires the calibration of $G_{eb}$ (shear modulus of the elastomeric support) and $F_{ya}$ (yield force). In contrast, the second approach, AEB-model 2, which uses *elastomericBearingBoucWen* element, demands the calibration of six parameters, listed in Table 7. 

**Figure 10:**
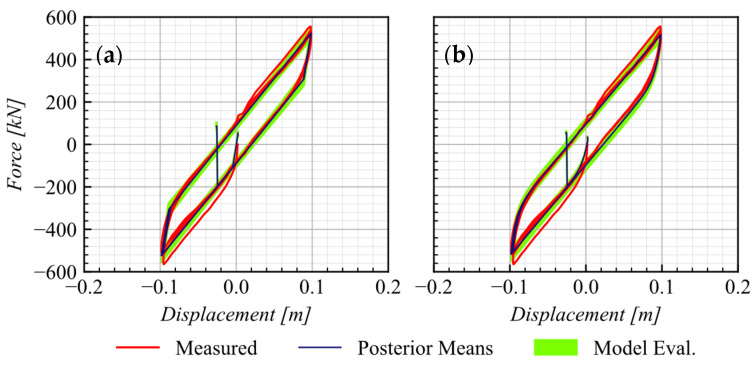
Comparison of the AEB from test series S1 with a calibrated response at 5th and 95th percentiles using (**a**) AEB-model 1 (**b**) AEB-model 2.

Figure 10 shows the excellent agreement between the measured responses from the AEB tests, as recorded in one test of series (see Table 3), and the predicted responses obtained using both constitutive models and posterior means and the 5th and 95th percentiles.

**Figure 11:**
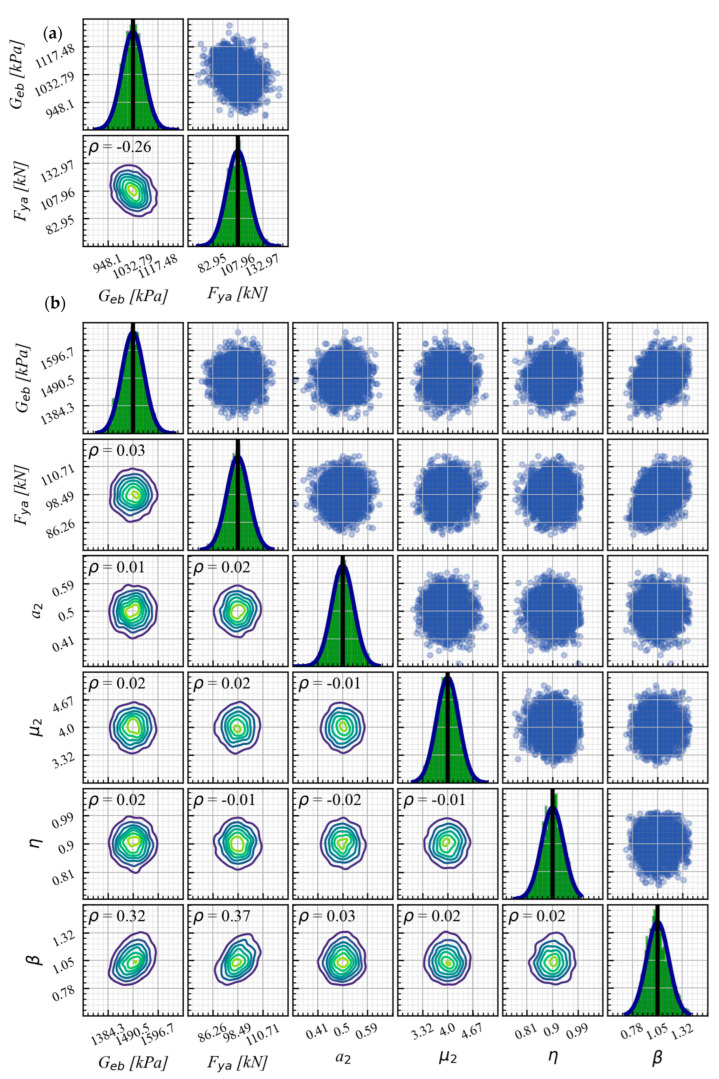
The AEB KDE matrix for test series S1 as a representative case. (**a**) AEB-model 1 and (**b**) AEB-model 2.

Figure 11a,b compares KDE matrices for the two modeling approaches representing the hysteretic behavior of AEB. It is worth noting that PDFs for $G_{eb}$ and $f_{ya}$, which were previously calibrated for AEB-model 1, are used as prior distribution for AEB-model 2 calibration. The results show that AEB-model 1 has a moderate negative correlation (−0.26) between $G_{eb}$ and $F_{ya}$, while AEB-model 2 has a negligible correlation (0.03) between the same pair of parameters. Additionally, AEB-model 2 shows moderate positive correlations among pairs $\beta -G_{eb}\;$(0.32) and $\beta -F_{ya}\;$(0.71), and the remaining parameters of the constitutive model exhibit mostly no correlations with values ranging from −0.01 to 0.03.

The difference in the correlation of the pair $G_{eb}-F_{ya}$ between AEB-model 1 and AEB-model 2 can be attributed to the number of parameters used in each constitutive model. AEB-model 1 uses only two parameters, while AEB-model 2 uses six. The increased number of parameters in AEB-model 2 improves the numerical modeling of the hysteretic behavior of AEB because it allows for better characterization of the different branches of the response of the EB.

**Figure 12:**
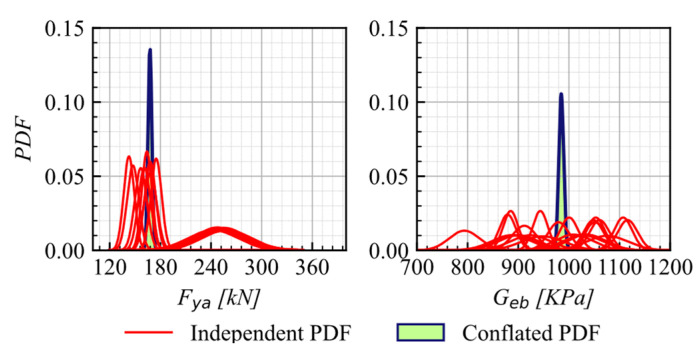
Conflated PDFs for $G_{eb}$ and $f_{ya}$ on AEB–model 1.

**Figure 13:**
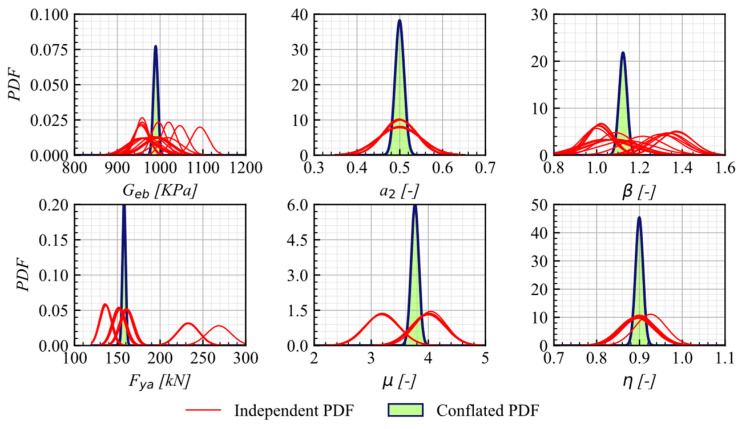
Conflated PDF for $G_{eb}$, $f_{ya}$, $a_{2}$, μ, β, and η on AEB-model 2.

Finally, the conflation of the PDFs for the independent calibrated parameters is applied to both approaches, resulting in the definitive PDFs for each parameter presented in Figure 12 and Figure 13.

### 5.2. Proposed PDFs

In summary, Table 8 presents a compilation of the joint PDFs for the constitutive model parameters of SB, UEB, and AEB, as representative structural components of highway bridges. These PDFs can be used to account for the uncertainty associated with the material constitutive models of these elements, thereby providing additional information to improve the accuracy of seismic response analysis for highway bridges. 

## 6. Conclusions

In this paper, data from comprehensive experimental campaigns on bridge components SBs, UEB, AEB, led by Rubilar (2015), Martinez (2017), and from tests on bearings from actual projects, are used to conduct Bayesian updating of key parameters of constitutive models characterizing the hysteretic response of these components. 

The PDFs of model parameters are obtained from independent tests conducted on the components. Then, these independent PDFs are merged in a single posterior PDF for each parameter by means of a conflation methodology, where the mean and COV are obtained for each key parameter. The Bayesian updating methodology implemented through SMC led to getting the KDE matrix, where the correlation between calibrated parameters is computed. In the particular case of AEB, the Bayesian estimation is carried out by means of two approaches: (a) an elasto-plastic approach (AEB-model 1) with two parameters to be calibrated and (b) a Bouc–Wen approach (AEB-model 2) using the *elastomericBearingBoucWen* element, which provides a better characterization of the hysteretic behavior of AEB, but in turn, requires the calibration of six parameters. 

The main contribution of the implementation of the Bayesian updating methodology for these components is the quantification of uncertainty obtained as a result of and expressed by means of posterior PDFs. The proposed joint PDFs for the governing constitutive model parameters of SB, UEB, and AEB can be considered to conduct detailed nonlinear FE analyses of bridges accounting for the model parameter uncertainty in the predicted responses with the use of the well-calibrated model parameters for bridge components.

It is noted that proposing PDFs for constitutive models of bridge components based on experimental data allows for the incorporation of the estimation results and the proper propagation of the associated uncertainties in state-of-the-art nonlinear finite element models of bridges. This is a crucial aspect to provide a more realistic representation of the complex seismic behavior of bridges, which is critical for ensuring the safety and resilience of transportation infrastructure and enhancing findings from new bridge design and analysis methodologies, such as PBSD. Finally, this research highlights the potential of Bayesian updates in bridge engineering and contributes to the development of more advanced and sophisticated design and analysis methodologies for bridges. The proposed methodology has broad implications for other types of engineering systems that involve significant uncertainty and variability, where improved accuracy and reliability are critical for safe and effective design and analysis.

## Figures and Tables

**Table 1:** Test specimens of SBs used in this study.

Specimen	*h_l_* (cm)
WD1/WD2	10
WOD1/WOD2	72

**Table 2:** Testing speed for each UEB specimen used in this study.

Test Tag	Cyclic Test Velocity (mm/s)
B1	25
B2	25
B3	10
B4	50
B5	75
B6	100

**Table 3:** Database of anchored elastomeric bearings.

Test Series	N	Width (mm)	Length (mm)	Diameter (mm)	Total Height (mm)
S1	5	650	650	-	98
S2	4	-	-	500	100
S3	6	320	320	-	53
S4	4	700	700	-	194
	4	1000	1000	-	230
S5	1	585	600	-	168

**Table 4:** Values of $\gamma_{i}$ as proposed by Martinez et al. (2017) [9].

Type of Seismic Bar	$\gamma_{1}$	$\gamma_{2}$
SBs-WD	0.04	0.71
SBs-WOD	0.07	0.31

**Table 5:** Bouc–Wen dimensionless parameters.

Parameter	
Post-yield stiffness ratio of non-linear hardening component	$a_{2}$
Exponent of non-linear hardening component	μ
Yield exponent	β
First hysteretic shape parameter	η

**Table 6:** Prior PDF for calibration of bridge components.

	Parameter	Distribution	Mean	COV (%)
SBs-WD (WD2)	$f_{y}$ (MPa)	Normal	235.46	5.82
	$g_{1}$(-)		0.11	36.36
SBs-WOD (WOD1)	$f_{y}$ (MPa)	Normal	266.24	8.40
	$g_{1}$(-)		0.09	22.22
	$g_{2}$ (-)		0.39	10.26
UEB (B1-C2)	$G_{eb}$ (kN/m^2^)	Normal	1083.60	3.60
	μ (-)		0.31	2.59
AEB—model 1 (S1)	$F_{y_{a}}$ (kN)	Normal	108.00	11.10
	$G_{eb}$ (kN/m^2^)		1033.50	2.5
AEB—model 2 (S1)	$F_{y_{a}}$ (kN)	Normal	98.45	4.89
	$G_{eb}$ (kN/m^2^)		994.00	3.10
	$a_{2}$		0.50	7.98
	μ		4.00	7.50
	β		0.90	4.33
	η		1.05	11.41

**Table 7:** Parameters of element *elastomericBearingBoucWen*.

Parameter	
Yield force	$F_{ya}$
Shear modulus of the bearing	$G_{eb}$
Post-yield stiffness ratio of non-linear hardening component	$a_{2}$
Exponent of non-linear hardening component	μ
Yield exponent	η
First hysteretic shape parameter	β

**Table 8:** PDFs’ calibrated parameters for SBs and EBs.

Component	Parameter	Distribution	Mean	COV (%)
SBs-WD	$f_{y}$ (MPa)	Normal	206.4	6.76
	$g_{1}$(-)		0.104	27.6
SBs-WOD	$f_{y}$ (MPa)	Normal	264.4	7.60
	$g_{1}$(-)		0.091	13.8
	$g_{2}$ (-)		0.395	6.25
UEB	$G_{eb}$ (kN/m^2^)	Normal	1176	1.15
	μ (-)		0.230	0.80
AEB-model 1	$f_{y_{a}}$ (kN)	Normal	167.6	1.72
	$G_{eb}$ (kN/m^2^)		985	0.39
AEB-model 2	$f_{y_{a}}$ (kN)	Normal	157.8	1.19
	$G_{eb}$ (kN/m^2^)		990	0.50
	$a_{2}$		0.50	2.0
	μ		3.765	1.74
	β		1.122	1.60
	η		0.899	1.00

## Data Availability

The data presented in this study are available on request from the corresponding author.
